# Complete Genome Sequence of Probiotic Strain *Lactobacillus acidophilus* La-14

**DOI:** 10.1128/genomeA.00376-13

**Published:** 2013-06-20

**Authors:** Buffy Stahl, Rodolphe Barrangou

**Affiliations:** DuPont Nutrition and Health, Madison, Wisconsin, USAa; Department of Food, Bioprocessing and Nutrition Sciences, North Carolina State University, Raleigh, North Carolina, USAb

## Abstract

We present the 1,991,830-bp complete genome sequence of *Lactobacillus acidophilus* strain La-14 (SD-5212). Comparative genomic analysis revealed 99.98% similarity overall to the *L. acidophilus* NCFM genome. Globally, 111 single nucleotide polymorphisms (SNPs) (95 SNPs, 16 indels) were observed throughout the genome. Also, a 416-bp deletion in the LA14_1146 sugar ABC transporter was identified.

## GENOME ANNOUNCEMENT

Several *Lactobacillus acidophilus* strains have been extensively characterized functionally to document their probiotic attributes (1–4). *In vivo* and *in vitro* studies of *L. acidophilus* strain La-14 have documented probiotic functionalities, including tolerance to gastrointestinal conditions, oxalate-degradation capability, bacteriocin production, and beneficial modulation of the immune response (5–7). The whole-genome sequence for La-14 was determined by FLX Titanium 454 sequencing (8). A total of 833,107 reads were assembled using the *L. acidophilus* NCFM genome as a template using NGen (DNASTAR, Madison, WI), and were subsequently inspected for quality using SeqMan Pro (DNASTAR, Madison, WI), resulting in an average coverage of 149×. The four gaps in the draft sequence were closed by PCR followed by Sanger sequencing. The La-14 and NCFM strains were determined to have a single coding sequence (CDS) for 23S rRNA at position 1.63 Mbp, though two had previously been annotated. Genomic annotations were assigned automatically by RAST (9), with some minor additions. The La-14 genome was subsequently aligned against previously sequenced complete genomes of *L. acidophilus* strains NCFM and 30SC (1, 10) and the *L. acidophilus* ATCC 4796 genome draft (69 contigs) using progressiveMauve (11).

Alignment of *L. acidophilus* genomes demonstrated that strains La-14 and NCFM are extremely similar and share high synteny with that of strain ATCC 4796, whereas notable differences were observed with strain 30SC. The overall G+C content of La-14 and NCFM are both 34.7%, which is close to that of ATCC 4796 (33.8%), whereas that of strain 30SC is 38.1%. A comparative analysis of the clustered regularly interspaced short palindromic repeats (CRISPRs) array revealed that this typically hypervariable locus was identical in La-14 and NCFM, and similar sequences were observed in ATCC 4796 (though the draft contains a gap), whereas this locus is absent in 30SC (a type I restriction-modification system is encoded at the equivalent position).

A global pairwise comparison of the highly similar genomes of La-14 and NCFM revealed a single 416-bp deletion in LA14_1146, within an ABC transporter ATP binding protein, homologous to LBA1131 in NCFM. Additional analysis of the differences between NCFM and La-14 revealed 16 single-base-pair indels, of which 14 predictively cause frameshifts. Of the 95 single-nucleotide polymorphisms (SNPs) discovered, 47 of them are nonsynonymous, and 29 occur in intergenic regions. A total of 52 genes are possibly affected by these minor changes.

The European Food Safety Authority (EFSA) stipulates that microbial strains used in food applications must not harbor acquired antimicrobial resistance genes to clinically relevant antimicrobials (12). *L. acidophilus* La-14 was phenotypically tested using the ISO 10932|IDF 223 method of minimum inhibitory concentration (MIC), and the results were measured against the microbial breakpoints set by the EFSA (12, 13). This strain does not possess any measure of antibiotic resistance that exceeds the breakpoints, and therefore, does not constitute an antibiotic resistance transfer risk. The La-14 genome showcases the similarities between the commercial strains of *L. acidophilus* sequenced thus far, and it provides an interesting opportunity to investigate how small genomic changes may influence the probiotic functionalities within this species.

### Nucleotide sequence accession number.

The complete genome sequence of *L. acidophilus* La-14 has been deposited in GenBank under the accession no. CP005926.
